# Ethical Challenges and Strategies in Implementing Social Assistive Robots in Dementia Care

**DOI:** 10.1093/geroni/igaf122.1546

**Published:** 2025-12-31

**Authors:** Jing Wang, Sajay Arthanat, Momotaz Begum, Dain LaRoche

**Affiliations:** University of New Hampshire, Durham, New Hampshire, United States; University of New Hampshire, Durham, New Hampshire, United States; University of New Hampshire, Durham, New Hampshire, United States; University of New Hampshire, Durham, New Hampshire, United States

## Abstract

The integration of social assistive robots (SARs) in dementia care presents both opportunities and challenges in enhancing support for persons living with dementia (PLWD) and their care partners. From the initial stages of development through deployment, this study highlights how ethical considerations were embedded in the interdisciplinary collaboration between researchers from engineering, health sciences and nursing to ensure SARs align with the needs, values, and autonomy of PLWD. Drawing on data from baseline and follow-up interviews, participant-robot interaction observations, and interdisciplinary discussions, we examine how ethical awareness shaped SAR design, functionality, and implementation strategies. Key ethical considerations included balancing autonomy with safety in SAR interventions, addressing privacy concerns related to smart sensing technologies, and ensuring transparency in decision-making. Findings reveal that co-designing SAR care protocols with PLWD and their care partners through person-centered design fostered trust and engagement. Adaptations based on user feedback allowed for greater responsiveness, ensuring that SAR interactions remained meaningful and respectful of individual preferences. Additionally, strategies such as user-driven customization, ongoing ethical oversight, and structured feedback loops reinforced participant agency while mitigating potential biases in technology deployment. This study underscores the critical role of interdisciplinary collaboration in navigating the ethical complexities of SAR integration in dementia care. By prioritizing ethical vigilance throughout development and deployment, SARs can be implemented in a manner that upholds dignity, inclusivity, and person-centered care, ultimately enhancing their effectiveness in supporting aging in place.

